# Developing and evaluating a predictive model for neonatal hyperbilirubinemia based on *UGT1A1* gene polymorphism and clinical risk factors

**DOI:** 10.3389/fped.2024.1345602

**Published:** 2024-02-29

**Authors:** Zhaoyang Cui, Wensheng Shen, Xuetong Sun, Yan Li, Ying Liu, Zhiyong Sun

**Affiliations:** ^1^ Department of Toxicology, School of Public Health, Jilin University, Changchun, China; ^2^ Department of Neonatology, Jilin Women and Children Health Hospital, Changchun, China; ^3^ NHC Key Laboratory of Radiobiology, School of Public Health, Jilin University, Changchun, China

**Keywords:** neonatal hyperbilirubinemia, predictive model, *UGT1A1* gene polymorphism, clinical risk factors, transcutaneous bilirubin measurements

## Abstract

**Background:**

Neonatal hyperbilirubinemia (NHB) is one of the most common diseases in the neonatal period. Without timely diagnosis and treatment, it can lead to long-term complications. In severe cases, it may even result in fatality. The *UGT1A1* gene and clinical risk factors play important roles in the development and progression of NHB.

**Methods:**

In this study, we conducted a cohort study and analyzed 3258 newborns from the Jilin Women And Children Health Hospital in northern China, including 372 children with hyperbilirubinemia. We established a predictive model using a logistic regression model based on clinical risk factors and the polymorphism of the G211A locus in the *UGT1A1* gene of newborns. Furthermore, the performance of the prediction model was evaluated using the ROC curve.

**Results:**

The logistic regression model indicates that the following factors are associated with an increased risk of NHB: the time when stool turns yellow [*P* ≤ 0.001, *OR* 1.266 (95% *CI*: 1.125-1.425)]; neonatal cephalohematoma [*P* ≤ 0.001, *OR* 33.642 (95% *CI*: 21.823-51.861)]; hemolytic disease of newborn [*P* ≤ 0.001, *OR* 33.849 (95% *CI*: 18.589-61.636)]; neonatal weight loss [*P* ≤ 0.001, *OR* 11.275 (95% *CI*: 7.842-16.209)]; neonatal premature rupture of membranes (PROM) history [*P* = 0.021, *OR* 1.422 (95% *CI*: 1.056-1.917)]; genetic polymorphism at the *UGT1A1* gene G211A locus. Gestational age is a protective factor [*P* ≤ 0.001, *OR* 0.766 (95% *CI*: 0.686-0.855)]. Compared to natural labor, cesarean section is a protective factor [*P* = 0.011, *OR* 0.711 (95% *CI*: 0.546-0.926)], while assisted delivery is a risk factor [*P* = 0.022, *OR* 2.207 (95% *CI*: 1.121-4.346)]. The area under the curve (AUC) of this prediction model is 0.804 (95% *CI*: 0.777-0.831), indicating good discrimination ability and value for predicting the risk of NHB after birth.

**Conclusion:**

We have developed and evaluated a predictive model that combines *UGT1A1* gene polymorphism and clinical risk factors for the first time. By using this nomogram and taking into account the results of serum total bilirubin measurement or transcutaneous bilirubin measurement, early prediction of the risk of neonatal hyperbilirubinemia can be achieved.

## 1 Introduction

Neonatal hyperbilirubinemia (NHB), characterized by elevated levels of bilirubin in the serum, is one of the most common clinical conditions affecting newborns, particularly preterm infants. It is also a leading cause of newborns requiring readmission to the hospital within the first week of their life (1, 2). Without timely diagnosis and treatment, bilirubin can traverse the blood-brain barrier trigger bilirubin encephalopathy and lead to long-term complications. In severe cases, it may even result in fatality (3–6). NHB and its subsequent condition, bilirubin encephalopathy, have consistently posed a significant disease burden worldwide (7, 8). In order to enhance the assessment and diagnosis of infants with hyperbilirubinemia, both the American Academy of Pediatrics (AAP) statement from 2022 and the 2014 consensus of experts on the diagnosis and treatment of NHB in China stress the significant importance of considering high-risk factors in the diagnosis of NHB (9, 10). Research from the 1970s indicated that the primary risk factors for NHB were infections. As the research progressed, it became evident that the incidence of NHB was associated with various factors such as premature infant, newborn weight loss, and breast feeding (11, 12). In recent years, researchers have incorporated more clinical-relevant factors into the analysis of risk factors for NHB, with the hope of identifying factors with stronger causal associations. This approach aims to provide greater clinical benefits when implementing targeted preventive measures against specific risk factors.

As the incidence of unexplained NHB gradually increases, some studies suggest that in the diagnosis and treatment of NHB, it is necessary to comprehensively consider clinical risk factors and genetic factors (13). Studies have been shown that many genes play an important role in the metabolism of bilirubin in newborn infants (14–17). Different genes have different functions, among which the *UGT1A1* gene, as the key enzyme responsible for conjugating bilirubin, is the only enzyme with the ability to glucuronidate bilirubin, playing the most critical role in the bilirubin metabolic pathway (18, 19). Polymorphisms in the *UGT1A1* gene can occur in coding exons, promoters, distal enhancer sequences, introns, and splice sites (20). Most genetic variations can reduce the function or activity of *UGT1A1* enzyme, which may lead to the occurrence of NHB, such as G211A and promoter TATA box (21). G211A mutation leads to a reduction in *UGT1A1* enzyme activity by approximately 70% (22). The normal sequence of TATA box is A(TA)_6_TAA, and the most common variation involves the insertion of an extra TA sequence into the TATA box, creating A(TA)_7_TAA. The more TA repeat sequences there are, the lower the transcriptional activity of *UGT1A1* becomes (23). However, the relationship between the genetic polymorphism of different loci and the incidence of NHB in different ethnic groups is still controversial, especially the variation of the promoter TATA box.

Therefore, by collecting data from newborns born at the Jilin Women and Children Health Hospital in China, the aim of this study is to further elucidate the impact of genetic variations, specifically the G211A and TATA box polymorphisms of the UGT1A1 gene, on the incidence of NHB in northern China. Additionally, this research endeavors to investigate the relationship between clinical factors, UGT1A1 gene polymorphisms, and NHB prevalence in the Chinese population, emphasizing the combined role of environmental and genetic factors in the diagnosis of the condition. At the same time, a nomogram for the early diagnosis of NHB was constructed and evaluated based on this, providing a basis for the early diagnosis and prevention of NHB.

## 2 Materials and methods

### 2.1 Research design

This study is a cohort studay conducted at Jilin Women And Children Health Hospital in China. The study population consists of newborns born at the hospital between September 2021 and June 2023. The inclusion criteria for study participants comprised neonates who had undergone genotyping for both the G211A polymorphism and the TATA box polymorphism of the *UGT1A1* gene. The exclusion criteria encompassed neonates with substantial missing data, as well as those with congenital malformations or significant genetic diseases that could potentially confound the diagnosis of NHB. Clinical information of newborns and their mothers, as well as the genetic polymorphism results for the G211A and the TATA box, were collected from the hospital's medical records system. The study included a total of 3,258 newborns, out of which 372 were diagnosed with NHB, resulting in an incidence rate of 11.42%. The diagnosis of NHB in the study followed the criteria outlined in the Chinese consensus on the diagnosis and treatment of Neonatal hyperbilirubinemia (2014) (10), the specific operation was to measure the TcB value using a Japanese Minolta JM-103 transcutaneous bilirubin meter on a daily basis. Reference was made to the neonatal hour-specific bilirubin nomogram developed by Bhutani VK et al. in the United States. When the TcB value exceeded the 75th percentile of the nomogram, TSB was measured. When the TSB exceeded the 95th percentile of the nomogram, it was defined as hyperbilirubinemia. The study aims to analyze the relationship between clinical factors and *UGT1A1* gene polymorphisms and the incidence of NHB in China. A binary logistic regression model is utilized to investigate the impact of clinical factors and *UGT1A1* gene polymorphisms on the occurrence of NHB. This research has received approval from the hospital's ethics committee. Most of the newborns in the study were the Han nationality.

### 2.2 Detection of the G211A locus and the TATA box polymorphism in the *UGT1A1* gene

All neonates born at the Jilin Women And Children Health Hospital are advised by their doctors to undergo testing for the G211A locus and the TATA box polymorphism of the *UGT1A1* gene. The neonates' guardians are free to choose whether or not to proceed with the testing. If guardians choose to proceed, they are required to sign a consent form after being informed of the purpose and procedures of the testing. After 72 h of birth, the newborn will undergo blood collection. Following DNA extraction, PCR amplification and sequencing are performed to determine the polymorphisms of the *UGT1A1* gene at the G211A locus and the TATA box. This study solely focuses on the results of these polymorphisms in neonates.

### 2.3 Variable definitions

Hemolytic disease of newborn, refers to neonates with a positive direct Coomb's test were diagnosed with hemolytic disease. In this study, the ABO blood type and Rh blood type of the neonate and their mother were considered. Neonatal weight loss, refers to a decrease in neonatal weight of more than 10% of the birth weight after birth. Neonatal intrauterine distress history is defined as the occurrence of hypoxia and acidosis in the fetus during intrauterine development. Neonatal intrauterine distress is primarily caused by various high-risk factors in the mother, fetus, or placenta, and is characterized by abnormal fetal heart rate, a series of metabolic and reactive changes, and a comprehensive presentation that endangers the life and health of the neonate.Neonatal premature rupture of membranes (PROM) history is defined as the rupture of the fetal membranes prior to delivery. In this study, rupture of the fetal membranes more than 18 h before delivery is considered premature rupture of membranes. Maternal gestational hypertension history is defined as the occurrence of hypertension in the mother during pregnancy. Gestational hypertension usually appears after 20 weeks of gestation and is characterized by symptoms such as high blood pressure and proteinuria. Maternal gestational diabetes history is defined as the diagnosis of diabetes mellitus in the mother during pregnancy. Maternal prenatal infection history is defined as the occurrence of infectious diseases in the mother prior to delivery. Common prenatal infections include urinary tract infections, reproductive tract infections, and respiratory tract infections. The diagnosis of these diseases or disease histories was made by doctors in the Neonatology Department of Jilin Women And Children Health Hospital.

### 2.4 Statistical analyses

We conducted the analysis using BONC DSS Statistics 25.0 data analysis software. For quantitative variables that followed a normal distribution, we presented statistics using mean ± standard deviation. For non-normally distributed quantitative variables, we used the median and interquartile range. Qualitative variables were described using frequency and composition percentages. To compare two groups of quantitative variables, we utilized independent samples *t*-test for normally distributed data and the Mann-Whitney *U* test for non-normally distributed data. For comparisons involving three or more groups of quantitative variables, we applied analysis of variance (ANOVA). Two groups of qualitative variables were compared using either the chi-squared test or Fisher's exact probability test. Furthermore, we performed multifactor analysis of NHB using a binary logistic regression model to explore influencing factors. A nomogram was constructed based on the binary logistic regression model results in the R programming language. Model performance was evaluated using ROC curves and the area under the curve (AUC). We considered a *P*-value less than 0.05 as statistically significant.

## 3 Results

### 3.1 Relationship between quantitative and qualitative variables of neonates and the incidence of NHB

In order to explore the relationship between clinical factors and *UGT1A1* gene polymorphism and the incidence of NHB in Chinese newborns, the collected factors were categorized into quantitative and qualitative variables for analysis. In the quantitative variables, the gestation age of the neonates ranged from 28 to 42 weeks, with an average of 39.12 ± 1.11 weeks. The birth weight of the neonates ranged from 1,790 to 5,470 g, with an average of 3,399.43 ± 396.40 g. The discharge age of the neonates ranged from 1 to 15 days, with a median of 5 days and a interquartile range of 1 day, The numbers of gestational ranged from 1 to 7 times, with a median of 1 time and a interquartile range of 1 time. The parity ranged from 1 to 3 times, with a median of 1 time and a interquartile range of 0 times. The numbers of abortions ranged from 0 to 6 times, with a median of 0 times and a interquartile range of 1 time. The time of breastfeeding initiation ranged from 0 to 3 h, with a median of 0.5 h and a interquartile range of 0.7 h. The time to first meconium ranged from 0.5 to 28 h, with a median of 8 h and a interquartile range of 10 h.The time when stool turns yellow ranged from 1 to 9 h, with a median of 3 h and a interquartile range of 1 h. The average daily frequency of stools ranged from 0 to 10 times, with a median of 3 times and a interquartile range of 1 time. Table 1 presents the differences in the quantitative variables between the neonatal hyperbilirubinemia group and the non-hyperbilirubinemia group. There were significant differences observed between the hyperbilirubinemia group and the non-hyperbilirubinemia group in terms of newborn gestational age, newborn discharge age, and the time when stool turns yellow (*P *≤ 0.05). The results show that newborns with hyperbilirubinemia had a significantly lower mean gestational age (38.9 ± 1.1) compared to newborns without hyperbilirubinemia (39.2 ± 1.1, *P *≤ 0.001). Furthermore, the hyperbilirubinemia group had a longer duration of hospitalization (*P *= 0.007) and a longer time for the stool to turn yellow (*P *≤ 0.001). Table 2 illustrates the relationship between qualitative variables in clinical factors of newborns and their mothers and the occurrence of hyperbilirubinemia. Significant differences were observed between the hyperbilirubinemia group and the non-hyperbilirubinemia group in terms of maternal blood type, mode of delivery, neonatal cephalohematoma, hemolytic disease of newborn, neonatal weight loss, neonatal premature rupture of membranes (PROM) history, neonatal amniotic fluid status, and maternal gestational diabetes history. The incidence of hyperbilirubinemia in newborns with maternal blood types A (14.1%), B (10.8%), and O (17.4%) was significantly higher than in newborns with maternal blood type AB (8.7%) (*P *≤ 0.001). The rate of hyperbilirubinemia in newborns delivered through natural labor (14.7%) and assisted delivery (30.2%) was significantly higher than in newborns delivered by cesarean section (10.9%) (*P *≤ 0.001). Newborns with the following conditions, namely, neonatal cephalohematoma (68.7%), hemolytic disease of newborn (67.3%), neonatal weight loss (44.4%), and neonatal premature rupture of membranes (PROM) history (16.1%), exhibited significantly higher incidence rates of hyperbilirubinemia compared to newborns without these conditions (*P *≤ 0.001). For maternal gestational diabetes history, we found that a total of 799 study subjects had a diabetes history.The incidence of hyperbilirubinemia in newborns whose mothers had a history of diabetes during pregnancy (13.9%) was higher than in newborns whose mothers had no history of gestational diabetes (*P *= 0.043). Additionally, newborns with clear amniotic fluid (13.6%) had a significantly higher incidence of hyperbilirubinemia than newborns with turbid amniotic fluid (9.5%) (*P *= 0.028).

**Table 1:** Relationship between quantitative variables of neonates and the incidence of hyperbilirubinemia.

Factor	NH (*n *= 2,886)		H (*n *= 372)		*t/Z*	*P*
	M/Med	SD/IQR	M/Med	SD/IQR		
Gestational age (weeks)	39.2	1.1	38.9	1.1	4.928	0.001
Birth weight (g)	3,401.5	397.0	3,383.5	391.9	0.825	0.409
Age at discharge (days)	5.0	1.0	5.0	1.0	2.69	0.007
Numbers of gestational	1.0	1.0	1.0	1.0	0.49	0.625
Parity	1.0	0	1.0	0	0.89	0.373
Numbers of abortions	0	1.0	0	1.0	0.11	0.909
The time of breastfeeding initiation (hours)	0.5	0.7	0.5	0.6	1.17	0.204
The time to first meconium (hours)	8.0	10.0	8.0	9.0	0.12	0.904
The time when stool turns yellow (days)	3.0	1.0	3.0	0	3.99	0.001
Average daily frequency of stools	3.0	1.0	3.0	1.0	1.09	0.276

**Table 2:** Relationship between qualitative variables in newborns and the incidence of hyperbilirubinemia.

Factor		NH (*n *= 2,886) *n* (%)	H (*n *= 372) *n* (%)	$x^{2}$	*P*	*OR* (*95% CI*)
Gender of newborns	Male	1,462 (50.7)	188 (50.5)	0.002	0.965	1.005 (0.810–1.247)
	Female	1,424 (49.3)	184 (49.5)			
Singleton or twins	Singleton	2,855 (98.9)	371 (99.7)	1.447	0.229	0.248 (0.034–1.824)
	Twin	31 (1.1)	1 (0.3)			
Maternal ethnicity	Han Ethnicity	2,782 (96.4)	360 (96.8)	0.137	0.711	0.892 (0.486–1.637)
	Non-Han Ethnicity	104 (3.6)	12 (3.2)			
Maternal blood type	A Type	594 (20.6)	84 (22.6)	17.715	0.001	1.618 (1.013–2.584)
	B Type	1,292 (44.8)	139 (37.4)			1.231 (0.789–1.920)
	O Type	714 (24.7)	124 (33.3)			1.987 (1.256–3.119)
	AB Type	286 (9.9)	25 (6.7)			1.000
Mode of delivery	Natural labor	1,237 (42.9)	182 (48.9)	16.643	0.001	0.881 (0.719–1.079)
	Caesarean section	1,596 (55.3)	174 (46.8)			
	Assisted delivery	53 (1.8)	16 (4.3)			
Neonatal cephalohematoma		36 (1.2)	79 (21.2)	386.667	0.001	21.345 (14.137–32.230)
Hemolytic disease of newborn		18 (0.6)	37 (9.9)	172.559	0.001	17.598 (9.907–31.258)
Infant feeding method	Exclusive Breastfeeding	423 (14.7)	53 (14.2)	3.253	0.197	1.109 (0.951–1.294)
	Artificial Feeding	1,142 (39.6)	131 (35.2)			
	Mixed Feeding	1,321 (45.8)	188 (50.5)			
Neonatal weight loss		85 (2.9)	68 (18.3)	173.128	0.001	7.371 (5.246–10.357)
Neonatal resuscitation history		16 (0.6)	3 (0.8)	0.057	0.811	1.458 (0.423–5.029)
Neonatal intrauterine distress history		197 (6.8)	27 (7.3)	0.096	0.757	1.068 (0.704–1.622)
Neonatal premature rupture of membranes (PROM) history		528 (18.3)	101 (27.2)	16.587	0.001	1.664 (1.300–2.131)
Neonatal amniotic fluid status	Turbid	503 (17.4)	48 (12.9)	4.803	0.028	0.702 (0.511–0.969)
	Clear	2,383 (82.6)	324 (87.1)			
Maternal gestational hypertension history		148 (5.1)	21 (5.6)	0.179	0.672	1.107 (0.692–1.772)
Maternal gestational diabetes history		692 (24.0)	107 (28.8)	4.077	0.043	1.280 (1.007–1.628)
Maternal cholestasis history		12 (0.4)	2 (0.5)	0	1.000	1.295 (0.289–5.807)
Maternal prenatal infection history		31 (1.1)	7 (1.9)	1.230	0.267	1.766 (0.772–4.040)

### 3.2 Relationship between *UGT1A1* gene variants and NHB

To explore the relationship between *UGT1A1* gene polymorphism and NHB. We primarily observed the genetic polymorphism results at two loci of the *UGT1A1* gene, namely, the G211A polymorphism and the promoter TATA box (Table 3). The results demonstrate that genetic polymorphism at the G211A locus significantly increases the incidence of hyperbilirubinemia in newborns (*P *= 0.011). The incidence of hyperbilirubinemia is significantly higher in newborns with heterozygous and homozygous mutations at the *UGT1A1* gene G211A locus (13.7%) compared to those with the wild type (10.2%) (*P *= 0.003). The variation in the TATA box does not significantly impact the incidence of NHB (*P *= 0.136). Joint analysis of both loci reveals that the incidence of hyperbilirubinemia in newborns with genetic polymorphism is higher than those wild type.

**Table 3:** Relationship between *UGT1A1* gene variants and hyperbilirubinemia.

*UGT1A1* gene variants		NH (*n *= 2,886) *n* (%)	H (*n *= 372) *n* (%)	$x^{2}$	*P*	*OR* (*95% CI*)
c.211 G > A variant	GG	1,882 (65.2)	213 (57.3)	9.082	0.011	1.000
	GA	929 (32.2)	147 (39.5)			1.398 (1.117–1.750)
	AA	75 (2.6)	12 (3.2)			1.141 (0.756–2.643)
	GA + AA	1,004 (34.8)	159 (42.7)	9.081	0.003	1.399 (1.124–1.742)
TATA box polymorphism	TA_6_/TA_6_	2,198 (76.2)	300 (80.6)	3.997	0.136	1.000
	TA_6_/TA_7_	659 (22.8)	70 (18.8)			0.778 (0.592–1.024)
	TA_7_/TA_7_	29 (1.0)	2 (0.5)			0.505 (0.120–2.128)
	TA_6_/TA_7_ + TA_7_/TA_7_	688 (23.8)	72 (19.3)	3.705	0.054	0.767 (0.585–1.006)
c.211 G > A variant combine TATA box polymorphism	No genetic variant	1,371 (47.5)	160 (43.0)	4.129	0.127	1.000
	One genetic variant	1,338 (46.4)	193 (51.9)			1.236 (0.989–1.544)
	Tow genetic variants	177 (6.1)	19 (5.1)			0.920 (0.558–1.518)

### 3.3 Relationship between *UGT1A1* gene polymorphism and transcutaneous bilirubin measurements

To further investigate the impact of genetic polymorphism at the G211A and TATA box loci on NHB in China, we collected transcutaneous bilirubin measurement values from the second to fifth day after birth. All newborns had their transcutaneous bilirubin values collected on the second day after birth. Due to factors like discharges and loss to follow-up, we were unable to collect transcutaneous bilirubin values for all newborns on the third to fifth day. Nonetheless, we analyzed the differences in transcutaneous bilirubin values from second to fifth day between different genotypes at the two loci and when both loci were jointly analyzed for the entire population, the non-hyperbilirubinemia group, and the hyperbilirubinemia group. We found that in the entire population and the non-hyperbilirubinemia group, significant differences in transcutaneous bilirubin values on the fourth day were observed between different genotypes at the two loci (Table 4). However, no differences were observed in the hyperbilirubinemia group. In the total population and non-hyperbilirubinemia group, the transcutaneous bilirubin levels of newborns with genotype AG and AA were significantly higher than those with genotype GG (*P *≤ 0.001), the transcutaneous bilirubin measurement values of newborns with genotypes TA_6_/TA_7_ and TA_7_/TA_7_ were significantly lower than those of A_6_/TA_6_ (*P *≤ 0.03). When both loci were jointly analyzed, newborns with the presence of any genetic polymorphism or both genetic polymorphisms had higher transcutaneous bilirubin measurement values compared to newborns without genetic polymorphism (*P *≤ 0.02). The above differences were not found in newborns in the hyperbilirubinemia group.

**Table 4:** Relationship between *UGT1A1* gene polymorphism and the fourth day transcutaneous bilirubin measurements (M ± SD).

Genotype	General population (*n* = 3,066)	NH (*n* = 2,720)	H (*n* = 346)
GG	10.6 ± 2.6	10.1 ± 2.3	14.6 ± 2.3
GA	11.2 ± 2.6	10.6 ± 2.1	14.7 ± 2.2
AA	11.5 ± 2.7	10.9 ± 2.0	15.5 ± 3.4
*F*	18.922	16.132	0.634
*P*	0.001	0.001	0.531
TA_6_/TA_6_	10.9 ± 2.6	10.4 ± 2.3	14.6 ± 2.3
TA_6_/TA_7_	10.6 ± 2.6	10.1 ± 2.1	15.1 ± 2.3
TA_7_/TA_7_	10.2 ± 2.2	9.9 ± 2.0	14.4 ± 0.5
*F*	3.849	3.824	1.501
*P*	0.021	0.022	0.224
No genetic variant	10.6 ± 2.6	10.2 ± 2.3	14.4 ± 2.3
One genetic variant	11.0 ± 2.6	10.4 ± 2.2	14.9 ± 2.2
Tow genetic variants	10.8 ± 2.4	10.4 ± 2.0	14.7 ± 2.7
*F*	6.845	3.983	1.536
*P*	0.001	0.019	0.217

### 3.4 Development and evaluation of NHB risk prediction model

**Figure 1:**
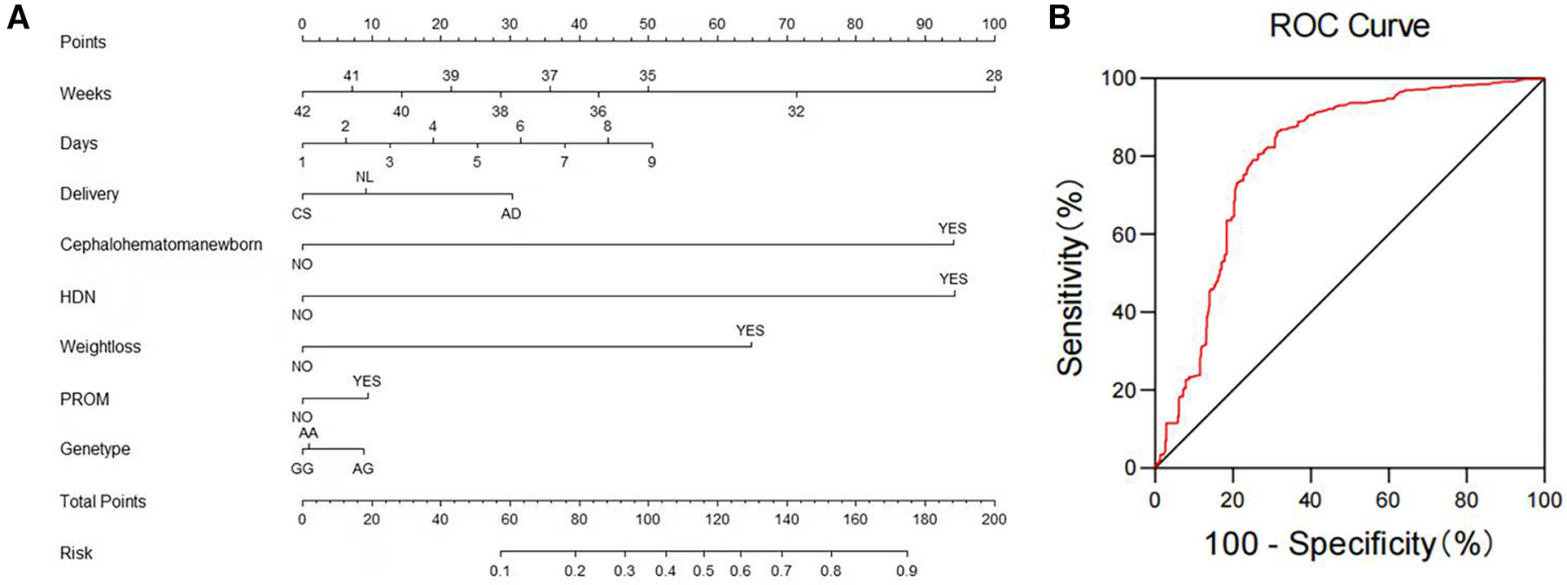
NHB risk prediction nomogram and ROC curve based on risk factor analysis results. (**A**) Nomogram: The nomogram presents factors contributing to the risk prediction of newborn hyperbilirubinemia. These factors include neonatal gestational age (Weeks), the time when stool turns yellow (Days), mode of delivery (CS, Caesarean Section; NL, Natural labor; AD, Assisted Delivery), neonatal cephalohematoma, hemolytic disease of the newborn (HDN), neonatal weight loss, neonatal premature rupture of membranes history (PROM), and the G211A locus polymorphism of the *UGT1A1* gene (Genetype). (**B**) ROC Curve of the Risk Factor Analysis Model for Newborn Hyperbilirubinemia: This ROC curve represents the performance of the risk factor analysis model for newborn hyperbilirubinemia.

We utilized binary logistic regression models to analyze the risk factors for NHB (Table 5). The model indicates that the following factors are associated with an increased risk of NHB: the time when stool turns yellow [*P *≤ 0.001, *OR* 1.266 (*95% CI*: 1.125–1.425)]; neonatal cephalohematoma [*P *≤ 0.001, *OR* 33.642 (*95% CI*: 21.823–51.861)]; hemolytic disease of newborn [*P *≤ 0.001, *OR* 33.849 (*95% CI*: 18.589–61.636)]; neonatal weight loss [*P *≤ 0.001, *OR* 11.275 (*95% CI*: 7.842–16.209)]; neonatal premature rupture of membranes (PROM) history [*P *= 0.021, *OR* 1.422 (*95% CI*: 1.056–1.917)]; genetic polymorphism at the *UGT1A1* gene G211A locus. Gestational age is a protective factor [*P *≤ 0.001, *OR* 0.766 (*95% CI*: 0.686–0.855)]. Compared to natural labor, cesarean section is a protective factor [*P *= 0.011, *OR* 0.711 (*95% CI*: 0.546–0.926)], while assisted delivery is a risk factor [*P *= 0.022, *OR* 2.207 (*95% CI*: 1.121–4.346)]. Figure 1 presents a nomogram and the ROC curve of the NHB risk prediction model based on the analysis of risk factors. The nomogram (Figure 1A) includes factors such as neonatal gestational age (Weeks), the time when stool turns yellow (Days), mode of delivery (Delivery, CS, Caesarean Section; NL, Natural labor; AD, Assisted Delivery), neonatal cephalohematoma (Cephalohematoma), hemolytic disease of the newborn (HDN), neonatal weight loss (Weightloss), neonatal premature rupture of membranes history (PROM), and genetic polymorphism at the G211A locus (Genetype). Each line on the nomogram is scaled according to the range of values for each factor. The length of each line reflects the contribution of that factor to the risk of NHB. By adding up the scores for each factor, a total score is obtained, and using this total score, a vertical line is drawn to estimate the corresponding probability of developing hyperbilirubinemia. The ROC curve is used to evaluate the binary logistic regression model (Figure 1B). The points on the ROC curve represent different cut-off points. The area under the ROC curve (AUC) indicates the model's ability to distinguish between patients and non-patients. A larger AUC suggests a stronger ability to discriminate and more accurate predictions. In this model, the AUC is 0.804(*95% CI*: 0.777–0.831), indicating that the model has good discrimination ability and is valuable for predicting the risk of NHB after birth.

**Table 5:** Analysis of risk factors for neonatal hyperbilirubinemia.

Factor		*Β*	SE	*χ^2^*	*df*	*P*	Adjusted OR (95% CI)
Gestational age (weeks)		−0.267	0.056	22.574	1	0.001	0.766 (0.686–0.855)
The time when stool turns yellow (days)		0.236	0.060	15.234	1	0.001	1.266 (1.125–1.425)
Mode of delivery	Natural labor						1.000
	Caesarean section	−0.341	0.135	6.405	1	0.011	0.711 (0.546–0.926)
	Assisted delivery	0.792	0.346	5.250	1	0.022	2.207 (1.121–4.346)
Neonatal cephalohematoma		3.516	0.221	253.479	1	0.001	33.642 (21.823–51.861)
Hemolytic disease of newborn		3.522	0.306	132.646	1	0.001	33.849 (18.589–61.636)
Neonatal weight loss		2.423	0.185	171.069	1	0.001	11.275 (7.842–16.209)
Neonatal premature rupture of membranes (PROM) history		0.352	0.152	5.358	1	0.021	1.422 (1.056–1.917)
c.211 G > A variant	GG						1.000
	AG	0.330	0.134	6.114	1	0.013	1.391 (1.071–1.808)
	AA	0.032	0.391	0.007	1	0.935	1.032 (0.480–2.221)

## 4 Discussion

This study conducted a analysis of the clinical factors in neonates and the results of genetic polymorphism at two loci of the G211A and the TATA box, to investigate the relationship between these factors and NHB. The results demonstrate that both genetic variations and clinical factors can influence the susceptibility to NHB. Factors such as the time when stool turns yellow, neonatal cephalohematoma, hemolytic disease of newborn, neonatal weight loss, neonatal premature rupture of membranes history and genetic polymorphism at the *UGT1A1* gene G211A locus were identified as risk factors for NHB in Chinese newborns. Gestational age was found to be a protective factor, whereas cesarean section was protective compared to natural labor, and assisted delivery was identified as a risk factor. In addition, we found that the incidence of NHB in this study was as high as 11.42%, further emphasizing the need for the identification of risk factors for NHB in China. After that, we used clinical risk factors and *UGT1A1* gene polymorphism results to construct a prediction model of NHB. Daunhawer I et al. (24) constructed a prediction model of NHB using clinical risk factors, which suggested that it can improve the early diagnosis of NHB, minimize unnecessary bilirubin measurement in low-risk neonates and avoid the occurrence of bilirubin encephalopathy in high-risk neonates. Unlike them, we first introduced genetic polymorphism results into the process of constructing the nomogram. We hope to further improve the accuracy of the prediction model. However, we must acknowledge that in our model, the role of *UGT1A1* gene G211A locus polymorphism in the early diagnosis of NHB is relatively minor compared to other factors. Therefore, further studies are still needed to assess the broader applicability of genetic polymorphisms in NHB.

In this study, apart from the well-established risk factors such as lower gestational age, neonatal cephalohematoma, hemolytic disease of newborn, and neonatal weight loss, we discovered that the time when stool turns yellow is a risk factor for hyperbilirubinemia. Stool passage is the primary means by which neonates eliminate bilirubin from their bodies. Delayed passage of meconium or a prolonged time when stool turns yellow can exacerbate neonatal jaundice. If jaundice symptoms progressively worsen, it can potentially lead to hyperbilirubinemia (25). Furthermore, we found that among the modes of delivery, natural labor and assisted delivery are risk factors for NHB, while cesarean section may serve as a protective factor. This may be because neonates are more likely to experience pressure or stretching of the birth canal during natural labor or assisted delivery. Such pressure or stretching can lead to vascular bleeding or cephalohematoma in neonates, increasing the likelihood of postnatal hyperbilirubinemia. Alkan S (26) and Anthony E (27). have also suggested that cesarean section can result in a decrease in neonatal jaundice. It is undeniable that there is still some controversy about the relationship between cesarean section and neonatal hyperbilirubinemia. After all, some indications of cesarean section for neonates may affect the results, such as macrosomia. Therefore, we still need to further explore the relationship between cesarean section neonates and hyperbilirubinemia in a larger data set by controlling confounding factors. Additionally, in this study, we found that neonatal premature rupture of membranes history is a high-risk factor for hyperbilirubinemia. Premature rupture of membranes in neonates may increase the chances of fetal infection, hypoxia, and liver dysfunction, consequently causing or exacerbating jaundice (28). Finally, in the results of the univariate analysis, we observed significant differences in some factors between the hyperbilirubinemia group and the non-hyperbilirubinemia group. Firstly, newborns with mothers of blood types A, B, and O had a significantly higher incidence of hyperbilirubinemia compared to newborns with mothers of blood type AB. This may be attributed to the fact that when the mother's blood type is AB, she lacks anti-A or anti-B antibodies in her serum, making it less likely to cause hemolytic reactions with the newborn's red blood cells, thereby reducing the risk of hyperbilirubinemia (29). Secondly, newborns with a maternal history of gestational diabetes had a higher incidence of hyperbilirubinemia. The results of Blasi I et al. are similar to ours (30).

In the analysis of *UGT1A1* gene mutations, the genetic polymorphism at the G211A locus is clearly identified as a risk factor for hyperbilirubinemia in Chinese newborns. When compared to developed regions, the elevated prevalence of NHB observed in China or within this study suggests that testing for polymorphisms at the UGT1A1 gene G211A locus in high-risk areas may offer significant potential value and benefits for the early diagnosis of NHB. A meta-analysis conducted by Mehrad-Majd H et al. (21) similarly found that the G211A gene polymorphism is a risk factor for hyperbilirubinemia in Asian neonates, and it suggested that the genetic polymorphism at the G211A locus has the potential to become an early diagnostic method for NHB and may serve as a marker for predicting the severity of jaundice. In this study, the genetic polymorphism at the TATA box locus did not exhibit statistically significant effects on the incidence of hyperbilirubinemia in Chinese neonates. In recent years, there has been some controversy regarding the relationship between genetic polymorphism at the TATA box locus and NHB. Halis H et al. (31) found that genetic polymorphism at the *UGT1A1* gene TATA box locus is a risk factor for severe hyperbilirubinemia in Turkish neonates. However, other studies have found no correlation between genetic polymorphism at this locus and NHB (32–35). Some research has even suggested that genetic polymorphism at the TATA box locus can reduce the risk of neonatal jaundice and act as a protective factor (36, 37). Therefore, further research with a larger dataset or basic research results is needed to delve deeper into the relationship between TATA box and the incidence of NHB, as well as to elucidate the underlying mechanisms. This will help clarify the relationship between TATA box polymorphism and NHB in different ethnic groups.

In summary, the occurrence of NHB in Chinese newborns is influenced by a combination of clinical factors and *UGT1A1* genetic polymorphisms, particularly neonatal gestational age, the time when stool turns yellow, mode of delivery, neonatal cephalohematoma, hemolytic disease of the newborn, neonatal weight loss, neonatal premature rupture of membranes history, and the genetic mutation at the G211A. Based on this, we obtained a nomogram. By utilizing this nomogram and combining serum total bilirubin measurements or transcutaneous bilirubin measurements, it is possible to identify newborns at risk of future hyperbilirubinemia at an earlier stage. This enables timely clinical observation and intervention, ultimately reducing the disease burden associated with NHB and its potential long-term effects.

### 4.1 Limitations

There are certain limitations to this study. Firstly, the sample size is limited, and the development of the prediction model was conducted in a single cohort. Therefore, the generalizability of the study results has certain limitations. Secondly, unfortunately, there were cases of neonates being discharged or lost to follow-up, which led to incomplete collection of transcutaneous bilirubin values on the third to the fifth days. Thirdly, in our study, the diagnosis of NHB was based on the hour-specific bilirubin nomogram developed by Bhutani VK et al. However, it is important to note that the accuracy of this nomogram may be limited as it did not consider the impact of ethnicity and other high-risk factors. This limitation underscores the necessity of exploring risk factors for NHB within different ethnic groups. Finally, this study used transcutaneous bilirubin measurements rather than conjugated bilirubin measurements, let alone unconjugated bilirubin measurements that can penetrate the neonatal blood-brain barrier and have direct neurotoxicity. These shortcomings have some limitations in explaining the impact of *UGT1A1* gene polymorphism on the incidence rate of NHB in China, especially the impact of TATA box polymorphism. We believe that with the deepening of research and technological progress, the impact of more clinical risk factors and gene polymorphisms on NHB will become increasingly clear.

## 5 Conclusion

We have developed and evaluated a predictive model that combines *UGT1A1* gene polymorphism and clinical risk factors for the first time. By using this nomogram and taking into account the results of serum total bilirubin measurement or transcutaneous bilirubin measurement, early prediction of the risk of neonatal hyperbilirubinemia can be achieved.

## Data Availability

The original contributions presented in the study are included in the article/Supplementary Material, further inquiries can be directed to the corresponding authors.
