# Supplementary material for: Developing and evaluating a predictive model for neonatal hyperbilirubinemia based on UGT1A1 gene polymorphism and clinical risk factors
Source: Front Pediatr. 2024 Feb 29;12:1345602. doi: 10.3389/fped.2024.1345602 (PMC10937529; doi:10.3389/fped.2024.1345602)
Supplement: Supplementary file 1 [file Datasheet1.pdf]

Supplementary-Developing and evaluating a predictive model for neonatal hyperbilirubinemia based on UGT1A1 gene polymorphism and clinical risk factors

Table S1. Relationship Between UGT1A1 gene polymorphism and the second day transcutaneous bilirubin measurements (M±SD)

Genotype	General population(n=3254)	NH (n=2882)	H (n=372)
GG	4.8±1.7	4.7±1.6	6.1±2.0
GA	4.9±1.6	4.7±1.6	5.8±1.7
AA	5.1±1.7	4.8±1.4	6.5±2.4
F	1.180	0.767	1.962
P	0.307	0.464	0.142
TA_6/TA_6	4.9±1.7	4.8±1.6	6.0±1.9
TA_6/TA_7	4.7±1.6	4.6±1.5	6.0±2.0
TA_7/TA_7	4.6±1.3	4.5±1.3	6.0±0.6
F	4.896	4.651	0.027
P	0.008	0.010	0.973
No genetic variant	4.9±1.7	4.7±1.6	6.1±2.0
One genetic variant	4.9±1.6	4.7±1.5	6.0±1.9
Tow genetic variants	4.7±1.6	4.5±1.5	5.9±1.9
F	1.502	1.290	0.664
P	0.223	0.276	0.515

Table S2. Relationship Between UGT1A1 gene polymorphism and the third day transcutaneous bilirubin measurements (M±SD)

Genotype	General population(n=3251)	NH (n=2879)	H (n=372)
GG	8.4±2.2	8.1±2.0	10.8±2.4
GA	8.6±2.1	8.3±1.9	10.5±2.0
AA	9.0±2.04	8.6±1.7	11.2±2.6
F	5.441	4.276	1.395
P	0.004	0.014	0.249
TA_6/TA_6	8.6±2.2	8.3±2.0	10.6±2.3
TA_6/TA_7	8.3±2.1	8.1±1.8	11.0±2.4
TA_7/TA_7	7.9±1.8	7.8±1.8	9.5±0.9
F	4.024	3.597	0.871

Genotype	General population(n=3251)	NH (n=2879)	H (n=372)
P	0.018	0.028	0.420
No genetic variant	8.5±2.2	8.2±2.0	10.7±2.4
One genetic variant	8.6±2.1	8.2±1.9	10.7±2.2
Tow genetic variants	8.4±2.0	8.2±1.7	10.6±2.5
F	0.985	0.358	0.067
P	0.374	0.699	0.935

Table S3. Relationship Between UGT1A1 gene polymorphism and the fifth day transcutaneous bilirubin measurements (M±SD)

Genotype	General population(n=1784)	NH (n=1563)	H (n=221)
GG	11.4±3.0	10.8±2.5	16.5±1.7
GA	12.1±2.7	11.4±2.2	16.3±1.4
AA	12.7±2.2	12.1±1.6	16.6±1.6
F	14.773	16.596	0.330
P	0.001	0.001	0.179
TA_6/TA_6	11.8±2.9	11.1±2.4	16.4±1.6
TA_6/TA_7	11.4±2.8	10.8±2.3	16.5±1.7
TA_7/TA_7	11.2±2.9	10.5±2.3	16.2±0.9
F	2.976	1.751	0.096
P	0.051	0.180	0.909
No genetic variant	11.5±3.0	10.8±2.5	16.4±1.7
One genetic variant	11.9±2.8	11.2±2.3	16.4±1.6
Tow genetic variants	11.6±2.3	11.3±2.0	15.9±0.5
F	5.774	5.265	0.455
P	0.003	0.005	0.635
